# Vicariance Between *Cercis siliquastrum* L. and *Ceratonia siliqua* L. Unveiled by the Physical–Chemical Properties of the Leaves’ Epicuticular Waxes

**DOI:** 10.3389/fpls.2022.890647

**Published:** 2022-07-04

**Authors:** Rui F. P. Pereira, João Rocha, Paulo Nunes, Tânia Fernandes, Ajith P. Ravishankar, Rebeca Cruz, Mariana Fernandes, Srinivasan Anand, Susana Casal, Verónica de Zea Bermudez, António L. Crespí

**Affiliations:** ^1^Chemistry Department and Chemistry Centre, University of Minho, Braga, Portugal; ^2^CQ-VR, University of Trás-os-Montes e Alto Douro, Vila Real, Portugal; ^3^Herbarium and Botanical Garden, University of Trás-os-Montes e Alto Douro, Vila Real, Portugal; ^4^Department of Applied Physics, School of Engineering Sciences, KTH Royal Institute of Technology, Albanova University Centre, Stockholm, Sweden; ^5^LAQV-REQUIMTE, Department of Chemical Sciences, Faculty of Pharmacy, Laboratory of Bromatology and Hydrology, University of Porto, Porto, Portugal; ^6^Department of Chemistry, University of Trás-os-Montes e Alto Douro, Vila Real, Portugal; ^7^CITAB, Department of Biological and Environmental Engineering, University of Trás-os-Montes e Alto Douro, Vila Real, Portugal

**Keywords:** *Cercidoideae*, *Caesalpinoideae*, Mediterranean basin, epicuticular waxes, wettability, optical properties

## Abstract

Classically, vicariant phenomena have been essentially identified on the basis of biogeographical and ecological data. Here, we report unequivocal evidences that demonstrate that a physical–chemical characterization of the epicuticular waxes of the surface of plant leaves represents a very powerful strategy to get rich insight into vicariant events. We found vicariant similarity between *Cercis siliquastrum* L. (family *Fabaceae*, subfamily *Cercidoideae*) and *Ceratonia siliqua* L. (family *Fabaceae*, subfamily *Caesalpinoideae*). Both taxa converge in the Mediterranean basin (*C. siliquastrum* on the north and *C. siliqua* across the south), in similar habitats (sclerophyll communities of *maquis*) and climatic profiles. These species are the current representation of their subfamilies in the Mediterranean basin, where they overlap. Because of this biogeographic and ecological similarity, the environmental pattern of both taxa was found to be very significant. The physical–chemical analysis performed on the epicuticular waxes of *C. siliquastrum* and *C. siliqua* leaves provided relevant data that confirm the functional proximity between them. A striking resemblance was found in the epicuticular waxes of the abaxial surfaces of *C. siliquastrum* and *C. siliqua* leaves in terms of the dominant chemical compounds (1-triacontanol (C30) and 1-octacosanol (C28), respectively), morphology (intricate network of randomly organized nanometer-thick and micrometer-long plates), wettability (superhydrophobic character, with water contact angle values of 167.5 ± 0.5° and 162 ± 3°, respectively), and optical properties (in both species the light reflectance/absorptance of the abaxial surface is significantly higher/lower than that of the adaxial surface, but the overall trend in reflectance is qualitatively similar). These results enable us to include for the first time *C. siliqua* in the vicariant process exhibited by *C. canadensis* L., *C. griffithii* L., and *C. siliquastrum*.

## Introduction

**Figure 1:**
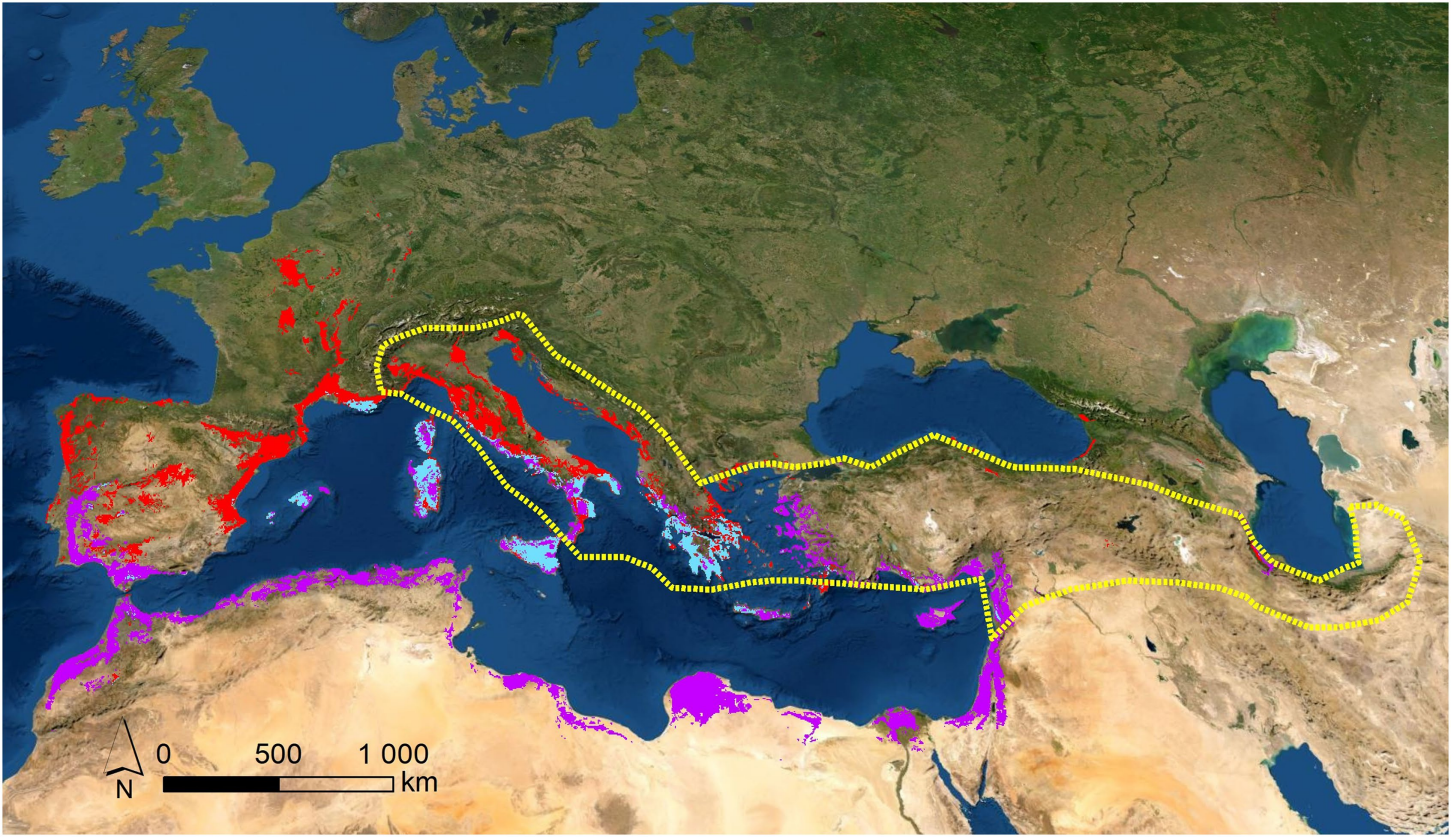
Potential (red areas) and real (area bordered with dotted yellow line) distributions for *C. siliquastrum*; potential distribution for *C. siliqua* (purple areas); and sympatric distribution for both species (light blue areas). These potential areas were obtained by SDM modelling, from herbaria and GBIF sources, previously confirmed (more details “Materials and Methods”).

In science, the holistic approach relies on a set of information originating from different analytical perspectives, which aim at contrasting and obtaining solid characterizations of systemic functionality (Schizas and Stamou, 2007). Holistic approaches are involved in the integration of multiple datasets for a better understanding of biological processes (Hervé et al., 2018). The major limitation of a holistic approach is the fact that any correlation with information provided by other established approaches depends critically on the quality and quantity of the data. The genus *Cercis* L. is a particularly good example to illustrate this aspect. *Cercis* is included in the family *Fabaceae*, subfamily *Cercidoideae* (Stevens, 2017). Ten species, located in arid and semi-arid regions of North America and Eurasia, encompass this genus. Fragmented distributions like these are typical of Arcto-Tertiary and Madrean-Tethyan geoflora dynamics (Axelrod, 1958; Milne and Abbott, 2002) with Eocenic origin (Davis et al., 2002; Fritsch and Cruz, 2012; Kozhoridze et al., 2015). The fragmentation of taxa explains the occurrence of the species across North America, Eastern Asia, and the Mediterranean basin. This is the reason why the closest taxa of the Mediterranean *C. siliquastrum* L. are *C. canadensis* L., present in the middle-East of the United States and South-East of Canada, and *C. griffithii* L., an Asiatic species isolated along Western Asia, (Li, 1944) with occurrence in West Iran (Sales and Hedge, 1996; Rezaipor et al., 2011; Sternberg, 2012). Phylogenetic evidences pointing out a common genetic flow across *C. siliquastrum*, *C. canadensis*, and *C. griffithii* were detected, despite the geographic distance between them (Geneve, 1991; Gang et al., 2001). For example, it was reported that the separation between *C. canadensis* and *C. siliquastrum* was concluded prior to the beginning of the Oligocene (Donoghue et al., 2001; Davis et al., 2002; Kadereit and Baldwin, 2012). In terms of habitat, the three species resemble closely. The distribution of *C. siliquastrum* is concentrated between Western Asia and South-Eastern Europe (Figure 1, red areas; Tutin, 1968; Al-Qaddi et al., 2017; Gianguzzi and Bazan, 2019; Doostmohammadi et al., 2020) where the ecological preferences are sclerophyllous shrubs (*maquis*) on calcareous rocks, (Pipinis et al., 2011; Gülsoy and Özkan, 2013; Sicuriello et al., 2014) similarly to *C. canadensis* and *C. griffithi* (Donselman and Flint, 1982; Abrams, 1994; Rhoades et al., 2005; Sternberg, 2012; Rezaipour et al., 2013; Ony et al., 2020). These phylogenetic, biogeographic, and ecological similarities are a manifestation of vicariant phenomena for these three species (Endler, 1982; Wiley, 1988). To guarantee the continuity of the biological process, (Craw et al., 1999) this vicariance relies on several other intermediate taxa of the same genus, with distributions spanning from Western North-America to Eastern and Central Asia (Thammina et al., 2017). The latter evidence is an additional proof demonstrating the common origin of the *Cercis* along the boreal hemisphere (Xiang et al., 1998, 2018; Ito et al., 2000; Garcia-Castaño et al., 2014).

**Figure 2:**
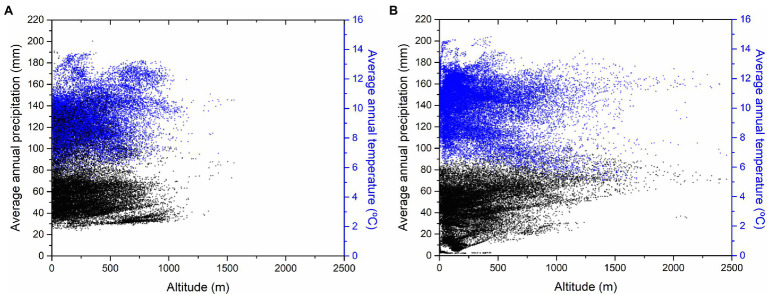
Altitudinal and thermopluviometric ranges for **(A)** *C. siliquastrum* **(B)** and *C. siliqua*. References were obtained from confirmed references for spontaneous occurrences – 21,370 references for *C. siliquastrum* (Global Biodiversity Information Facility, 2021a) and 25,518 for *C. siliqua* (Global Biodiversity Information Facility, 2021b).

The phylo-biogeographic and ecological dynamics of the above vicariant process could include other species and/or genera (Chen et al., 2014; Inda et al., 2014; Manafzadeh et al., 2014; Vargas et al., 2014; Mairal et al., 2017). In the present work, we hypothesized that *Ceratonia siliqua* L., another genus of the phylogenetic basic group of *Fabaceae* belonging to the subfamily *Caesalpinoideae*, with distribution in the Mediterranean basin, Northern Africa, and Western Asia, could be associated to the process, since it appears as a vicariant response of *C. siliquastrum* (Figure 1). In this region, these are the only two species of these subfamilies of *Fabaceae* to occur, and we suspected that this marked biogeographic and taxonomic proximity transforms *C. siliqua* into a promising candidate as a species of the same vicariant process. Previous molecular reports, as well as biogeographic and ecological characterizations, support the vicariant relationship between both species: (1) *Cercidoideae* show wider biogeographic distributions than *Caesalpinoideae*, probably because of the basal position of *Cercidoideae* in the systematics of *Fabaceae*; (Käss and Wink, 1996; Azani et al., 2017) (2) Several contacts are observed along the distributions of both subfamilies. Partial overlaps are detected for *C. siliquastrum* (Figure 1, red areas) and *C. siliqua* (Figure 1, purple areas) exclusively across the Mediterranean basin and Western Asia, with sympatric examples at the center and East part of this region (Figure 1, light blue areas), as a result of past wider potential overlapping; (Ramón-Laca and Mabberley, 2004) (3) As expected for taxa with such analogous biogeographic characteristics, *C. siliquastrum* and *C. siliqua* share very similar wild habitats—sclerophyllous shrubs (*maquis*; Ramón-Laca and Mabberley, 2004; Gülsoy and Özkan, 2013; Mahd et al., 2018)—and, not surprisingly, both species often grow in gardens all over the world; (4) The altitudinal and thermopluviometric ranges of both species; (Global Biodiversity Information Facility, 2021a,b) in their respective distribution areas are globally alike (Figure 2), although *C. siliquastrum* occupies more humid and temperate conditions, and *C. siliqua* prefers arid and dryer environments. In short, the marked biogeographic and climatic similarity discerned for *C. siliquastrum* and *C. siliqua* strongly suggests that both species may be addressed as ecologic and biogeographic vicariances.

Traditionally, eco-biogeography has been applied to describe vicariance dynamics (Wiley, 1988; van Veller et al., 1999). This has happened simply because geographical distribution is the most usual way to identify vicariance. Yet, under a generic concept like this, many other correlations can be considered. Morphologic, genetic or environmental platforms have been the most widely employed (Li and Christophel, 2000; Ali et al., 2019; Viruel et al., 2020). Taking into account that holistic characterizations are known to greatly benefit from the use of the widest possible set of different variables, and consequently from the largest possible number of analyses, the pursuit of distinct perspectives and characterization strategies is of the utmost interest to get deeper insight into dynamic vicariant systems. In this line of thought, based on several reports of environmental effects on the composition of epicuticular waxes, (Hull et al., 1979; Shepherd et al., 1995; Medina et al., 2004; Domínguez et al., 2011; Ortúñez and Cano-Ruiz, 2013; Rajčević et al., 2014; Menzel et al., 2017; Xue et al., 2017; Sharma et al., 2018) as well as on the close link established between the characteristics of epicuticular waxes of many taxa and taxonomic, ecological or evolutive issues, (Li and Christophel, 2000; Versieux et al., 2012; Li et al., 2016; Mitić et al., 2016; Harris et al., 2017; Klimko et al., 2018; Lindelof et al., 2020; Weber and Schwark, 2020; Faria et al., 2021) we propose in this work an unprecedented strategy to contribute to the study of vicariant phenomena in plant science. This new strategy involves a comprehensive physical–chemical analysis of the surface of plant leaves that strengthens the more usual phylogenetic, biogeographic and ecological approaches.

**Figure 3:**
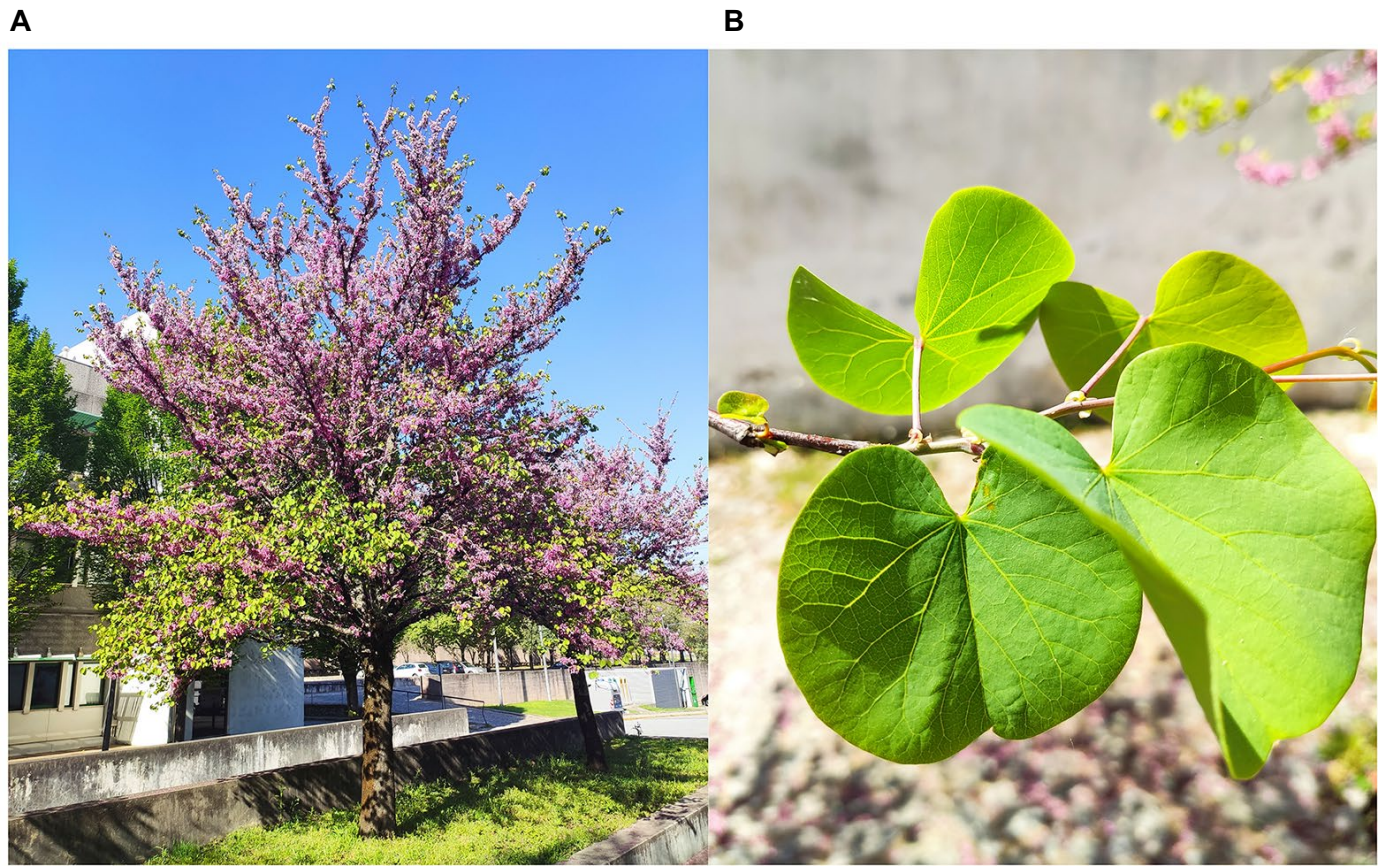
*C. siliquastrum* tree **(A)**, close view of leaves **(B)** showing the adaxial surface (front leaves) and abaxial surface (back leaves).

In this study, we demonstrate that a chemical- and physical-oriented characterization of the epicuticular waxes of the surface of plant leaves is an extremely valuable and straightforward approach to get rich insight into the vicariance biogeography existent between *C. siliquastrum* and *C. siliqua*, thus confirming the usefulness of this type of information in the framework of systematic characterizations (Engel and Barthlott, 1988). With this goal in mind, we have examined the chemical composition, morphology, and wettability of the epicuticular (and in some cases, also the intracuticular) waxes of the abaxial and adaxial surfaces of the *C. siliquastrum* leaf, and also the optical properties of the leaf (Figure 3). The results obtained have been subsequently compared with those reported by some of us recently for the *C. siliqua* leaf (Rodríguez et al., 2021).

We provide in this work clear proofs that the abaxial surfaces of the *C. siliquastrum* and *C. siliqua* leaves share a number of quite remarkable features that confirm their biological proximity: (a) the epicuticular waxes have the same dominant chemical compound (an alcohol whose length differs only in two carbon atoms), plate-like morphologies, and superhydrophobic behavior; and (b) very similar optical features. Our study also reveals that, in contrast to the abaxial surfaces, the adaxial surfaces of the leaves of these species retain in common plate-like morphology and similar optical properties, but differ significantly in terms of the dominant compounds (alkanes in *C. siliquastrum* and esters in *C. siliqua*) and wettability (*quasi* superhydrophobic in *C. siliquastrum* and *quasi* hydrophobic in *C. siliqua*). These observations suggest different biological strategies of the two species, which are manifested in their own characteristic geographical distributions.

## Materials and Methods

### Environmental Characterization

To deduce the ecological profiles of the *C. siliquastrum* and *C. siliqua* species two procedures were adopted: the calculation of the potential and real distributions, and the altitudinal and thermopluviometric characterization. To determine the potential areas for the species we used Maxent software. Maxent estimates the probability distribution of a species occurrence based on environmental constraints (Phillips et al., 2006). It requires only species’ presence data and environmental variables in Geographic Information System (GIS) layers for the study area. This information was obtained from the herbaria data included in the Global Biodiversity Information Facility, 2021a,b confirmed as spontaneous occurrences (subspontaneous or naturalized references were excluded). We used Maxent (version 3.4.3.1), to estimate the probability of potential suitable habitat for species occurrence ranging from 0 to 1, where 0 and 1 are the lowest and highest probabilities, respectively. The output obtained from Maxent modelling (in ASCII format) was input into the ArcGIS software (version 9.2, ESRI, Redlands, California, United States) as floating-point grids (Townsend Peterson et al., 2007) and occurrence probability of the species at each site was mapped. Areas with a potential habitat suitability over 50% were defined. Using Raster to Point tool, potential habitat area maps were transformed into point features. With the Multi-Values to Point extraction tool, and using the climate data retrieved from Worldclim platform, (WorldClim, 2021) climate data were assigned to each point. The real current distribution for the species was derived using the records confirmed in herbaria databases and bibliography. The results of such calculations are shown in Figure 1. Following Rocha et al. (2018) the altitudinal and thermopluviometric information was obtained from the environmental matrix elaborated on the basis of the potential modelling described above. To this purpose, milliards of points were selected regularly across the distribution areas of both species, and the altitudinal information (in m), monthly temperature (in °C) and precipitation (in mm), as well as nineteen bioclimatic (BIO) indexes, were retrieved from Worldclim platform (Fick and Hijmans, 2017). The calculated average values per variable are represented in Figure 2. Based on the potential distribution for *C. siliquastrum* and *C. siliqua*, this environmental comparison was performed using 21,370 and 25,518 data points along their potential areas, respectively (Global Biodiversity Information Facility, 2021a,b).

### Leaves Collection and Handling

Leaves collection is a critical aspect of the present physical–chemical study. The essence of this sort of analysis is to perform the characterization of the cuticular waxes immediately after collection and in a systematic way. The literature is rich in examples demonstrating the importance of collecting fresh plant material at Botanical Gardens or University Campus, thus close to the laboratory premises where the characterization is carried out (Meusel et al., 2000; Buschhaus et al., 2007; Pieniazek et al., 2022).

Prior to all the measurements, fresh green mature leaves were collected exclusively in springtime, early in the morning, from a *C. siliquastrum* specimen growing at the Gualtar Campus of the University of Minho (Braga, Portugal) shown in Figure 3. All measurements were repeated on a regular basis at the University of Trás-os-Montes e Alto Douro (UTAD; Vila Real, Portugal), with leaves collected from a *C. siliquastrum* tree existent at the Botanic Garden of UTAD. We note that Vila Real is 104 km far from Braga. Leaf samples were collected at the same development stage, same altitude and same orientation, from the same branch. In some cases, we had to wait 12 months to collect more leaves under the same conditions to obtain replicas. Overall, this part of the work lasted 2 years.

Sample handling is another essential aspect of the physical–chemical method employed. In order to analyze undamaged wax coatings, samples were, neither collected on rainy days, nor washed with water or cleaned with an inert gas flow. Samples were transported in zipper-sealed plastic bags.

### Extraction of the Epicuticular and Intracuticular Waxes

The epicuticular and intracuticular waxes of the adaxial and abaxial surfaces of a *C. siliquastrum* leaf were extracted independently using the mechanical (cryo-adhesion) method proposed by Ensikat et al. (2000) Twenty fresh leaf samples of approximately 3 × 2 cm^2^ were gently cut with scissors.

To isolate the epicuticular wax four drops of glycerol (Sigma-Aldrich), used as cryo-adhesive embedding liquid, were first placed on the surface of a metal plate to form a single drop. The latter was then pressed gently over the leaf surface and the resulting set was submerged slowly in liquid nitrogen for 5 s. After this period of time, the leaf was lifted off the frozen glycerol drop in which the epicuticular wax remained embedded. After thawing, the epicuticular wax layer was captured on chloroform and transferred to glass tubes. The solvent was evaporated and the glass tubes were sealed.

To isolate the intracuticular wax the above process was repeated using the same leaf sample. The glycerol drops were carefully placed exactly in the same spots considered in the isolation of the epicuticular wax. The residues of both types of waxes were subject to chemical characterization.

### Chemical Characterization of the Epicuticular and Intracuticular Waxes

After cryo-adhesion, the internal standard (tetradecane, 80 μl, 500 ng ml^−1^) was added to cuticular residues that were then dissolved in chloroform. A volume fraction was then taken to dryness under a nitrogen stream and derivatized with bis-trimethylsilyltrifluoroacetamide (BSTFA, CF_3_C[=NSi(CH_3_)_3_]OSi(CH_3_)_3_) and pyridine (1:1 v/v) at 70°C for 30 min in order to convert the hydroxyl groups of the compounds into the corresponding trimethylsilyl (TMS) derivatives. To check the system performance, an alkane standard solution (C10-C40) was used on a regular basis. All the reagents and standards were purchased at Supelco (United States) and Sigma-Aldrich (St. Louis, United States). Chromatographic separation was performed with an HP5-MS column (30 m × 0.25 mm I.D. × 0.25 μm film thickness, Agilent J&W, United States), with helium as carrier gas, a 80–325°C temperature gradient, and injection at 280°C in the pulsed splitless mode. A mass detector (5977B MSD) was used for identification purposes, with electron ionization of 70 eV set in full scan mode (m/z 50–650). For quantification purposes, detection was switched to a Flame Ionization Detector (FID) set at 280°C. The results are expressed as a relative response of the chromatographic areas after normalization to the internal standard. Supplementary Figure 1 shows representative chromatograms of the abaxial and adaxial surfaces of the *C. siliquastrum* leaf. The compounds were identified based on available standards and computer matching the reference mass spectra of the MS NIST Library (Supplementary Table 2). The NIST Mass Spectral Library provides different factors to identify unknown compounds, such as the Match Factor and Reversed Match Factor (National Institute of Standards and Technology, 2008). The Match Factor compares selected peaks (m/z values and their relative intensities) with the compounds registered in the library. Its value should be close to 999. The Reverse Match Factor is obtained after eliminating peaks that do not exist in the library spectrum entry. These two factors should be as similar to each other as possible. In order to improve the accuracy of the identification, only those substances that provided a Match Factor higher than 700 were selected for data processing. Procedural blanks helped identifying bibenzyl, hexadecane, 1-monopalmitin and 1-monostearin as procedural interferences, while an Extractable- and Leachable-screening standard for gas chromatography (TraceCERT®, Sigma-Aldrich, United States) helped recognizing bis(2-ethylhexyl) phthalate, stearic acid, cis-13-docosenoamide, tris(2,4-di-tert-butylphenyl)phosphate, and palmitic acid as procedural and system contaminants. Therefore, these substances, together with glycerol and other phthalates (likely derived from the waxes extraction procedure), were not taken into account in total area calculations.

### Statistical Analyses of the Epicuticular and Intracuticular Waxes

The chemical outcomes are shown as average values and standard deviation from duplicate analysis of each sample. To compare the waxes profile from the different leaves’ sections, normal distribution of the residuals and the homogeneity of variances were evaluated by means of the Shapiro–Wilk’s (sample size <50) and the Levene’s tests, respectively. All dependent variables were subsequently studied using a one-way ANOVA, subjected or not to Welch correction, followed by Duncan’s or Dunnett’s T3 test, depending if the requirement of the homogeneity of variances was verified or not, respectively. Principal components analysis (PCA) was applied to emphasize variation and highlight strong patterns in a dataset according to the different waxes’ main classes. For this purpose, oblimin with kaiser normalization was chosen as the rotation method considering that dependent variables were highly correlated. Statistical analyses were performed at a 5% significance level, using SPSS software (version 26.0, IBM Corporation, New York, United States).

### Microstructural Characterization

The Scanning Electronic Microscopy (SEM) of the epicuticular wax layer of *C. siliquastrum* leaves were obtained at the International Iberian Nanotechnology Laboratory (INL) using a FEI Quanta 650 FEG microscope (manufacture year 2012). Prior to SEM analysis, the leaves were gently cut into 5 × 5 mm2 samples and transferred to the microscope sample holder. Images were acquired under low vacuum water pressure (200 Pa) at 5.0 kV and using a Peltier Cooling Stage (1°C), leading to an environment with a relative humidity of about 30%. Image measurements were performed using ImageJ software (Schneider et al., 2012).

The Atomic Force Microscopy (AFM) measurements were performed in oscillating mode using an AFM CSI Nano-Observer equipment (Scientec) and a super sharp Si HQ:NSC19/FORTA with frequency resonance of 60 kHz and a spring constant of 0.3 Nm^−1^. The data collected were analyzed using the Gwyddion 2.52 software.

### Wettability Characterization

**Figure 4:**
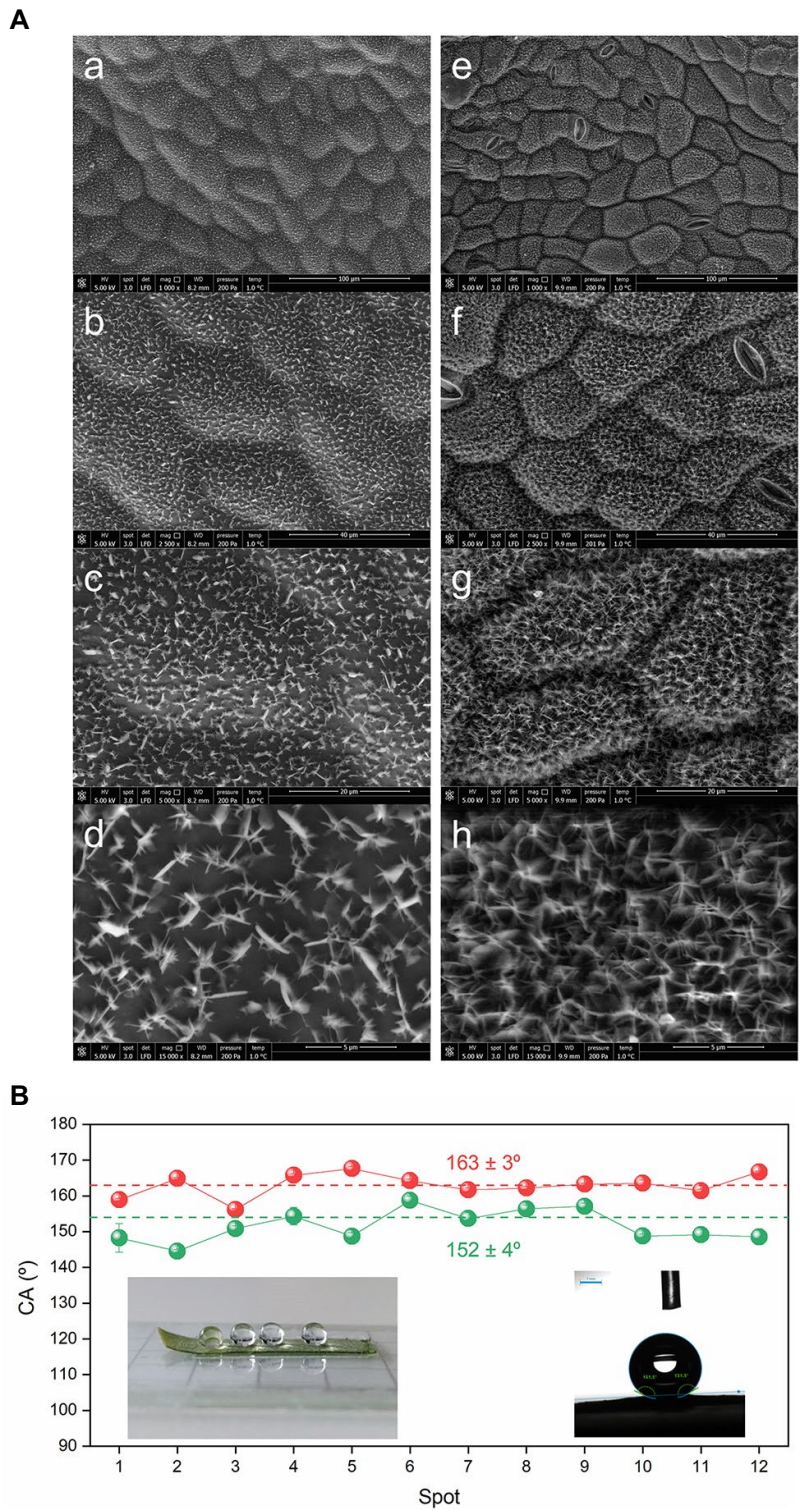
Morphology and wettability of the surfaces of the *C. siliquastrum* leaf. **(A)** SEM images at of the astomatous adaxial **(a–d)** and stomatous abaxial **(e–h)** surfaces. **(B)** Static water CA values of the adaxial (green symbols) and abaxial (red symbols) surfaces. Insets: drops of water sitting on the abaxial surface prior (left) and during (right) measurement.

The wettability of the adaxial and abaxial surfaces of the *C. siliquastrum* leaves was deduced by means of static measurements. The Contact Angle (CA) values were measured in a temperature-controlled chamber at 26 ± 1°C using a DSA25S drop shape analyzer (Krüss, Germany) controlled by the software ADVANCE. The volume of the water droplets was kept constant at 5 μl. Prior to measurement the diameter of the plastic needle used in the syringe was measured three times with an electronic micrometer and the average value was introduced in the software. The CA values were calculated from construction lines in digital images acquired by a video camera and using the Young-Laplace fitting. The contact line between the water drop and the leaf surface was placed manually by the operator, whereas the tangent line and the drop shape curve fit were both generated automatically by the software. Because leaf samples are never ideally flat, water drops usually sit behind a fold and lead to misleading values. To overcome this drawback, a clean, flat sample section was selected from each leaf (avoiding the border area), gently cut (0.5 × 2 cm^2^), and then carefully immobilized with double-faced adhesive tape in a glass slide. The resulting set was then fixed in the sample holder. The CA values were determined in a spot located at the middle of the sample surface (left inset of Figure 4B). At each spot 5 measurements were performed in 2 s interval. The results reported correspond to the average value of the measurements performed at each spot. The error analysis of the data was implemented by arithmetic mean of the root mean square error.

### Optical Characterization

The transmittance and reflectance (total and diffused) spectra of the adaxial and abaxial surfaces of the *C. siliquastrum* leaf were registered using a Perkin-Elmer Lambda 900 spectrophotometer with a 150 mm integrating sphere. A polarizer-depolarizer was used to ensure that the light interrogating the sample was unpolarized. The integrating sphere was used in transmittance and reflectance modes. Technical details of the two configurations adopted may be found elsewhere (Rodríguez et al., 2021). The measurements were performed on a *C. siliquastrum* leaf section (1 cm × 1 cm) cut from a set of around 4–6 leaves. A collimated light with a spot size of a few mm^2^ was used, making sure that the white light spot was incident on vein-free regions of the leaf. To check spectral reproducibility, 3 randomly-chosen sections from each leaf were examined. In the case of the angle resolved measurements, the leaf sample was placed at the center of the integrating sphere using a rotation mount and the total light (reflectance plus transmittance) inside the sphere was measured at different incident angles.

## Results

### Chemical Composition of the Epicuticular Waxes of the Surfaces of the *Cercis siliquastrum* Leaf

**Figure 5:**
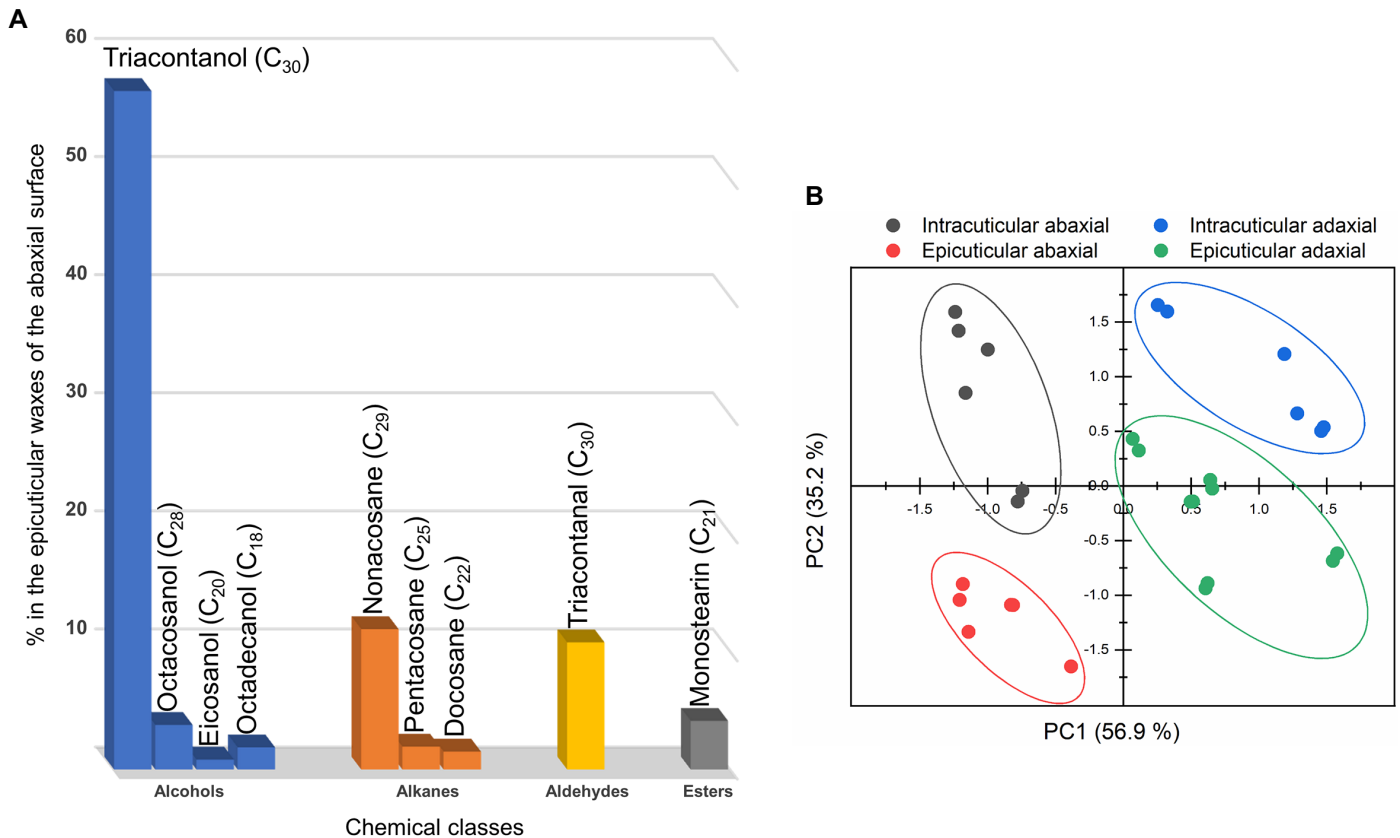
**(A)** Chemical classes and chemical compounds of the epicuticular waxes of the abaxial surface of the *C. siliquastrum* leaf. **(B)** Principal component analysis of the composition of the cuticular waxes: epicuticular abaxial (red symbols), intracuticular abaxial (grey symbols), epicuticular adaxial (green symbols) and intracuticular adaxial (blue symbols).

Based on the criteria adopted for the compounds’ identification (see Experimental section), 34 substances distributed along 4 main classes were identified in the epicuticular and intracuticular wax layers of the adaxial and abaxial surfaces of *C. siliquastrum* leaves. Figure 5A and Supplementary Table 1 reveal that the epicuticular waxes of the abaxial surface are dominated by alcohols (65.0%), with 1-triacontanol representing around 86% of this class. These coexist with alkanes (20.1%), aldehydes (10.8%), and esters (4.1%). The epicuticular wax layer of the adaxial surface of the *C. siliquastrum* leaf is mainly composed of high-chain linear alkanes (57.4%), alcohols (34.6%), esters (6.8%), and a minor proportion of squalene, a polyunsaturated hydrocarbon of the triterpenoid type (1.2%; Supplementary Table 1).

While the chemical classes of the adaxial and abaxial surface differ significantly, no statistical differences were observed between the epicuticular and intracuticular layers of each surface of the *C. siliquastrum* leaf in terms of the two major classes (i.e., alcohols and alkanes; Supplementary Table 1). However, for both surfaces marked differences were observed for minor substances, such as total esters (*p* = 0.02) and squalene (*p* < 0.001). The overall compositional dissimilarity can be responsible for the distinct hydrophobic behavior exhibited by the adaxial and abaxial surfaces of the leaf. The octanol/water partition coefficient (K_OW_) values and the relative chemical composition of the *C. siliquastrum* leaf (Supplementary Table 1) point out that the abaxial surface is considerably more hydrophobic (K_OW_ epicuticular ≈ 12.4 and K_OW_ intracuticular ≈ 12.0) than the adaxial surface whose layers demonstrate distinct hydrophobicity (K_OW_ epicuticular ≈ 9.1 and K_OW_ intracuticular ≈ 7.4).

We performed a PCA test to classify the sections of the *C. siliquastrum* leaf on the basis of its overall waxes’ composition. This analysis allowed us explaining 92.2% of the total data variance by using two principal components (Figure 5B), whose variable communalities were all higher than 0.835. Thus, 4 distinct groups were identified, corresponding to the two adaxial and two abaxial sections. The first principal component (PC1) factor (56.9% of the total variance) was able to discriminate two groups: the abaxial sections in the negative region, and the adaxial sections in the positive region. This result may be correlated with the fact that the abaxial waxes exhibited the highest values for alkanes (PC1 loading = 1.000) and a lower content of alcohols (PC1 loading = −0.973). The second principal component (PC2) factor represents only 35.2% of the total variance and was thus not significant.

### Morphology and Wettability of the Surfaces of the *Cercis siliquastrum* Leaf

The representative SEM images of the adaxial and abaxial surfaces of a *C. siliquastrum* leaf reproduced in Figure 4, show that the epicuticular waxes are organized in both cases as 1.3 ± 0.2 μm-long and 90 ± 20 nm-thick, randomly oriented plates, although their density is higher in the abaxial surface. Plates are thin, flat, frequently polygonal crystalloids, with distinct edges and variable size, which are attached to the surfaces at varying angles (Barthlott et al., 2008).

The topography of the latter surface was corroborated by Atomic Force Microscopy (AFM; Supplementary Figure 2).

Two examples of drops of water on the surface of a section of the abaxial surface of *C. siliquastrum* leaf are given in the insets of Figure 4B. Static water CA measurements reveal that the adaxial and abaxial surfaces can be classified as *quasi* superhydrophobic and superhydrophobic, respectively, as they lead to average CA values of 152 ± 4° (Figure 4B, green symbols) and 163 ± 3° (Figure 4B, red symbols), respectively. Their relative magnitude is in perfect agreement with the lower hydrophobic character of the adaxial surface with respect to the abaxial surface deduced from the K_OW_ values (9.1 and 12.4, respectively) derived above from the chemical data (Supplementary Table 1). We will return to this aspect below.

### Optical Properties of the *Cercis siliquastrum* Leaf

To characterize the reflectance, transmittance, and absorptance of the adaxial and abaxial surfaces of the *C. siliquastrum* leaf, we conducted visible–near infrared (vis–NIR) optical spectroscopy measurements that included the quantification of the total and diffused parts of the reflectance and total transmittance, and of the angle-dependent light absorptance.

**Figure 6:**
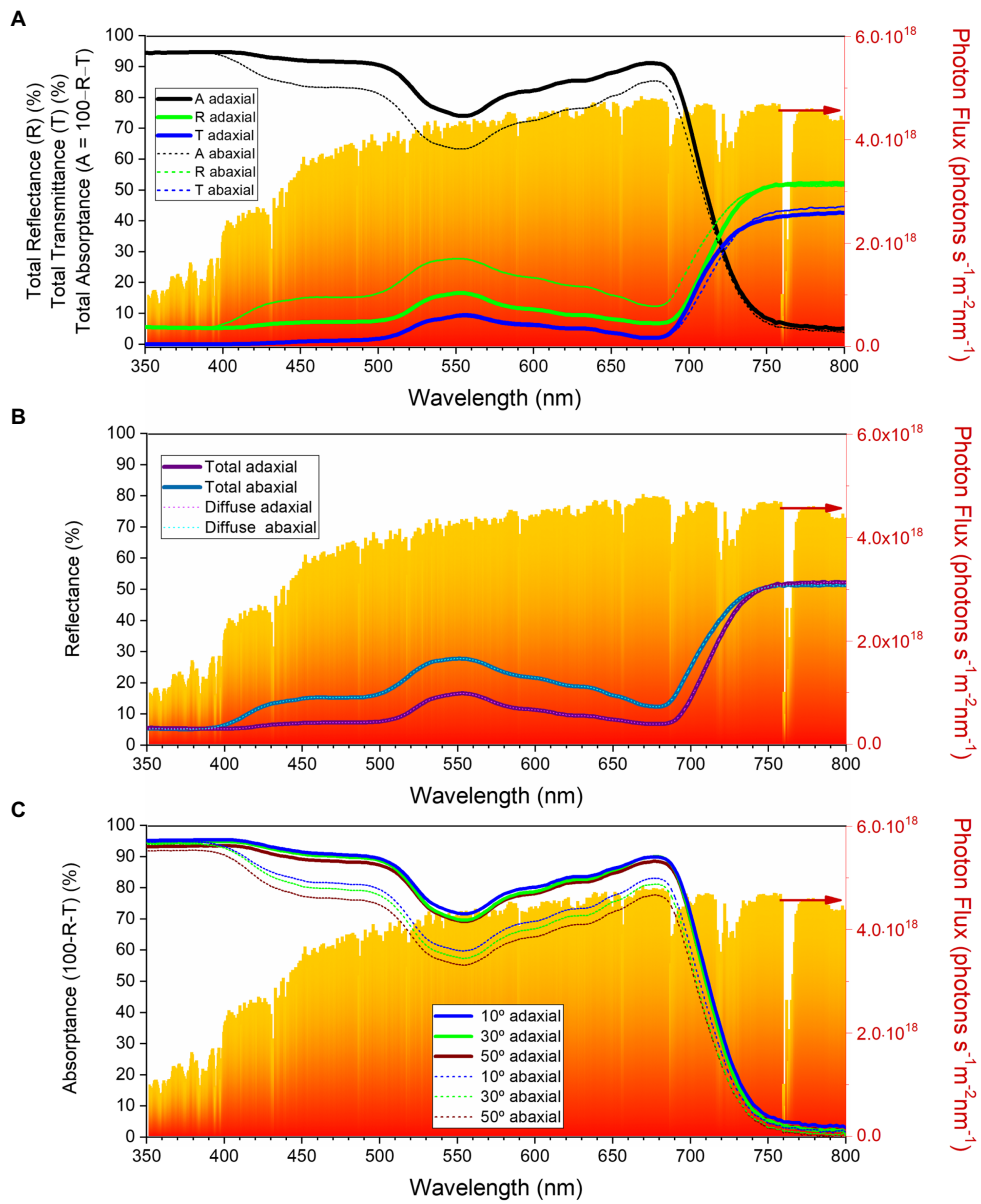
Optical characteristics of the *Cercis siliquastrum* leaf in the 350–800 nm wavelength range: **(A)** total reflectance (green lines), transmittance (blue lines) and absorptance (black lines) for the adaxial (thick lines) and abaxial (thin short dashed lines) surfaces; **(B)** total reflectance (dark thick lines) and diffused reflectance (light thin lines) for the adaxial (purple) and abaxial (blue) surfaces; **(C)** total absorptance as a function of incident angle for the adaxial (thick lines) and abaxial (thin lines) surfaces. The red-orange area in the graphs represents standard terrestrial solar spectra (AM1.5G).

Figure 6A describes the total transmittance (T), reflectance (R) and absorptance (A) measured for light incident on the adaxial and abaxial surfaces of a *C. siliquastrum* leaf in the 350–800 nm wavelength range. The total reflectance from the abaxial surface (dotted green line) is consistently higher than that of the adaxial surface (solid green line) in the 400–700 nm interval. This is particularly evident in the green region, i.e., around 550 nm, where the total reflectance of the abaxial surface (~28%) is 1.6 times higher than that of the adaxial side (~17%). Unlike the total reflectance, the total transmittance of the abaxial and adaxial surfaces differs only slightly over the entire wavelength region examined (Figure 6A, dotted and solid blue lines, respectively). The (total) absorptance spectra from the adaxial and abaxial surfaces of the *C. siliquastrum* leaf are shown in Figure 6A, solid and dotted black lines, respectively. The observed difference in absorptance is primarily due to the associated difference in reflectance from the adaxial and abaxial leaf surfaces.

The contribution of the diffused component to the total reflectance is depicted in Figure 6B. Clearly, the diffused reflectance dominates over the specular component on both surfaces of the *C. siliquastrum* leaf, pointing out lack of glossiness. We recall that in general the specular component of reflectance is negligible for near-normal light incidence (Grant et al., 1993). We also note that the internal tissues of the leaves are primarily responsible for the diffused reflectance.

To investigate how absorptance of light in the *C. siliquastrum* leaf varies with the incidence angle, we measured the sum of the reflectance and transmittance (R + T) by rotating the leaf kept at the center of an integrating sphere (Supplementary Figure 3). Absorptance is calculated as 1 − (R + T) and is shown on Figure 6C. These data show that the absorptance is weakly dependent on the angle of incidence. For example, around 550 nm, the absorptance of the adaxial surface varied only by 2–3% and that of the abaxial surface changed by 7–8%, as the angle of incidence increased from 10 to 50°. These observations suggest that changes in the sunlight angles, as well as in the position of the leaves, do not significantly affect light harvesting, which is not surprising.

## Discussion

Let us now compare the data obtained here for the *C. siliquastrum* leaf with those reported previously for the leaf of *C. siliqua* (Rodríguez et al., 2021).

The most noteworthy conclusion reached is definitely the extraordinary physical–chemical resemblance found between the abaxial surfaces of the leaves of both species: (1) The dominant chemical compound present in the abaxial epicuticular wax layers of these leaves (1-triacontanol in *C. siliquastrum* and 1-octacosanol in *C. siliqua*; Rodríguez et al., 2021) belong to the class of alcohols, and have practically the same molecular weight (C30 in *C. siliquastrum* and C28 in *C. siliqua* (Rodríguez et al., 2021)); (2) There is a marked morphological match between the epicuticular waxes of the abaxial surfaces which form a texture composed of an intricate network of randomly organized nanometer-thick and micrometer-long plates; (3) In both species the abaxial surfaces exhibit exactly the same superhydrophobic character, manifested by an essentially identical maximum water CA value (167.5 ± 0.5° in *C. siliquastrum* and 162 ± 3° in *C. siliqua* (Rodríguez et al., 2021)).

Comparison of the *C. siliquastrum* and *C. siliqua* leaves also allows inferring that the adaxial surfaces resemble closely from the standpoint of morphology and optical properties, but exhibit remarkable differences in terms of chemical composition and water retention ability: (1) The adaxial surfaces display both lower density and degree of interconnection of plates than the abaxial surfaces, an effect also found for other *Caesalpinioideae* taxa; (Neves et al., 2016; Mendes et al., 2021) (2) In both species the light reflectance/absorptance of the adaxial surface is significantly lower/higher than that of the abaxial surface, but the overall trend in the reflectance, the rise in the reflectance close to 550 nm, and the dip close to 700 nm, are qualitatively similar; (3) The optical response measured for the adaxial surface of the *C. siliquastrum* leaf shows higher reflectance and lower absorption in the 400–500 nm range than that of the *C. siliqua* leaf; (Rodríguez et al., 2021) (4) The dominant chemical compound present in the epicuticular layer of the adaxial surface of the *C. siliquastrum* leaf is 1-triacontanol, the same C30 alcohol prominent in the epicuticular layer of the abaxial surface (Supplementary Table 1). In contrast in the case of the *C. siliqua* leaf, the major compound present in the adaxial surface is 1-monopalmitin, (Rodríguez et al., 2021) a monocylglycerol with emulsion properties that may increase the water-holding capacity and thus wettability; (5) The epicuticular layer of the adaxial surface of *C. siliquastrum* features *quasi* superhydrophobic behavior (water CA of 152 ± 4° (Figure 4B, green symbols)), whereas that of *C. siliqua* demonstrates *quasi* hydrophobic properties (average water CA of 94 ± 8° (Rodríguez et al., 2021)).

The distinct wettability of the abaxial and adaxial surfaces of the leaves of *C. siliquastrum* and especially *C. siliqua* was reported for other species (Fernández et al., 2014; Koukos et al., 2015; Papierowska et al., 2018). Plant ecophysiological or ecohydrographical arguments were suggested to explain the differences observed. Typically, the abaxial surface exhibits higher hydrophobicity (higher CA value) than the adaxial surface (Kolyva et al., 2012). In the leaf, the abaxial surface plays the role of water management system, keeping the surface dry through its superhydrophobic nature, while allowing essential processes to occur (photosynthesis, respiration, transpiration, and evaporation). Moreover, the usually higher diffusive reflectance of the abaxial surface aids leaf cooling by reducing heating induced by absorption processes. The striking aspect discerned here is the large difference between the CA values of the abaxial and adaxial surfaces of the *C. siliqua* leaf (*ca.* 50°) which contrasts with the minor difference found for the *C. siliquastrum* leaf (*ca.* 10°). These findings are consistent with the reflectance/absorption differences measured for both surfaces for the two species. Such effects must be correlated with the fact that, as *C. siliqua* occurs in drier habitats than *C. siliquastrum*, especially along Northern Africa (Arabian-Saharian region), its adaxial surface is impelled to retain more water and thus be considerably less hydrophobic.

The set of results gathered for the abaxial surfaces of both species can be interpreted as unambiguous evidences of homology-based strategies (Müller, 2003). The histological response of *C. siliquastrum* is certainly an adaptation to the ecological restrictions imposed by the preferential wild habitats of the *Cercideae* (sclerophyllous shrubs or *maquis* of the Mediterranean basin and western Asia; Gonzalez Rodriguez et al., 2016). Similar responses were described for other *Fabaceae* exposed to environmental conditions where precipitation is concentrated over relatively short periods throughout the year, (Sanches and Válio, 2006; El-Ghazali, 2008; Bieras and Das Sajo, 2009; Dönmez and Aydin, 2018; Mendes et al., 2021) or even for other botanic families subject to analogous climatic conditions (Tomaszewski, 2004; Tschan and Denk, 2012; Kozhoridze et al., 2015; Eminagaoglu et al., 2020). Therefore, the present work confirms the existence of a direct correlation between the chemical composition, morphology, and wettability of the epicuticular waxes of plant leaves, and of the leaves’ optical properties, and the thermopluviometric restrictions imposed by their natural habitat.

The vicariance effect between *C. siliquastrum* and *C. siliqua* confirmed here, which is supported by the ecological similarity of their habitats, implies potential phylogenetic relationships. Both species are represented along different areas of the Mediterranean basin and Western Asia, but have very similar climatic profiles (Figure 2), in accordance with ancestral wider overlapping between them. Ecological and phylogenetic connections for both species confirmed this similarity (Sulaiman et al., 2003; Zhao et al., 2021). Thus, the physical–chemical information reported here provides additional fingerprints of the proximity between both species, in this case at the epicuticular waxes’ level. This vicariance has been essential to guarantee the presence of these two *Fabaceae* subfamilies along Western Eurasia.

In conclusion, we demonstrate for the first time that the chemical and physical characterization of the leaves’ epicuticular waxes is a useful, straightforward, and simple way of significantly improving the ecological description necessary to understand the phylo-biogeographic characterization of the two species of the basal subfamilies of *Fabaceae* examined here. This leads us to propose that the new approach illustrated here could be employed on a regular basis in the framework of other vicariant processes.

## Data Availability Statement

The original contributions presented in the study are included in the article/supplementary material, further inquiries can be directed to the corresponding authors.

## Author Contributions

All authors listed have made a substantial, direct, and intellectual contribution to the work and approved it for publication.

## Funding

This research was supported by National Funds from Foundation for Science and Technology (FCT) and FEDER through POCI-COMPETE 2020-Operational Programme Competitiveness and Internationalization in Axis I - Strengthening research, technological development and innovation (UID/QUI/00616/2013, UID/QUI/00616/2019, UIDB/04033/2020, UIDB/04033/2020, UID/QUI/50006/2020, PEST/QUI/00686/2020) and funded by project PORPLANTSURF - Superhydrophobic films inspired in the surface of plant leaves and petals from Northern Portugal (PTDC/CTM-REF/29785/2017; POCI-01-0145-FEDER-029785), financed by the European Regional Development Fund (ERDF) through COMPETE 2020 - Operational Program for Competitiveness and Internationalization (POCI) and FCT. AR and SA acknowledge partial support from the Swedish Energy Agency (grant no. 49227-1). PN acknowledges CQ-VR/FCT for a grant (UI/BD/151084/2021). RP and MF acknowledge FCT-UMinho and FCT-UTAD, respectively, for the contracts in the scope of Decreto-Lei 57/2016–Lei 57/2017.

## Conflict of Interest

The authors declare that the research was conducted in the absence of any commercial or financial relationships that could be construed as a potential conflict of interest.

## Publisher’s Note

All claims expressed in this article are solely those of the authors and do not necessarily represent those of their affiliated organizations, or those of the publisher, the editors and the reviewers. Any product that may be evaluated in this article, or claim that may be made by its manufacturer, is not guaranteed or endorsed by the publisher.

## Supplementary Material

The Supplementary Material for this article can be found online at: https://www.frontiersin.org/articles/10.3389/fpls.2022.890647/full#supplementary-material
