# Supplementary material for: Vicariance Between Cercis siliquastrum L. and Ceratonia siliqua L. Unveiled by the Physical–Chemical Properties of the Leaves’ Epicuticular Waxes
Source: Front Plant Sci. 2022 Jul 4;13:890647. doi: 10.3389/fpls.2022.890647 (PMC9289549; doi:10.3389/fpls.2022.890647)
Supplement: Supplementary file 1 [file Data_Sheet_1.docx]

Supplementary Material

Vicariance between *Cercis siliquastrum* L. and *Ceratonia siliqua* L. unveiled by the physical-chemical properties of the leaves’ epicuticular waxes

R. F. P. Pereira,^1*^ J. Rocha,^2,3^ P. Nunes,^2^ T. Fernandes,^1^ A. P. Ravishankar,^4^ R. Cruz,^5^ M. Fernandes,^2,6^ S. Anand,^4^ S. Casal,^5^ V. de Zea Bermudez,^2,6*^ A. L. Crespí^3,7*^

^1^Chemistry Department and Chemistry Centre, University of Minho, Braga, Portugal.

^2^CQ-VR, University of Trás os Montes e Alto Douro, 5000-811 Vila Real, Portugal.

^3^Herbarium and Botanical Garden, University of Trás-os-Montes e Alto Douro, 5000-811 Vila Real, Portugal.

^4^Department of Applied Physics, School of Engineering Sciences, KTH Royal Institute of Technology, Albanova University Centre, Roslagstullsbacken 21, SE-106 91 Stockholm, Sweden.

^5^LAQV‐REQUIMTE, Department of Chemical Sciences, Faculty of Pharmacy, Laboratory of Bromatology and Hydrology, University of Porto, 4050‐313 Porto, Portugal.

^6^Department of Chemistry, University of Trás-os-Montes e Alto Douro, 5000-811 Vila Real, Portugal.

^7^CITAB, Department of Biological and Environmental Engineering, University of Trás-os-Montes e Alto Douro, 5000-811 Vila Real, Portugal

*** Correspondence:**R.F.P. Pereira ([rpereira@quimica.uminho.pt](mailto:rpereira@quimica.uminho.pt)); V. de Zea Bermudez ([vbermude@utad.pt](mailto:vbermude@utad.pt)); A. L. Crespí ([acrespi@utad.pt](mailto:acrespi@utad.pt))

**Supplementary Table 1.** Relative amounts (%, mean + standard deviation) of the most abundant compounds in the cuticular waxes of the *C. siliquastrum* leaf.

| **Compound** | **Formula** | **chain length**  **C_n_** | **Log K_ow_^a^** | **abaxial** | |  | **adaxial** | |
| --- | --- | --- | --- | --- | --- | --- | --- | --- |
|  |  |  |  | **epicuticular** | **intracuticular** |  | **epicuticular** | **intracuticular** |
| 1-octadecanol | CH_3_(CH_2_)_n-1_OH | C_18_ | 7.7 | 1.9 ± 0.5 | 2.6 ± 0.8 |  | 4.9 ± 3.0 | 5.8 ± 5.0 |
| 1-eicosanol |  | C_20_ | 8.7 | 0.8 ± 0.7 | 3.4 ± 0.8 |  | 4.5 ± 2.3 | 6.8 ± 5.0 |
| 1-octacosanol |  | C_28_ | 12.6 | 3.8 ± 0.6 | 3.3 ± 0.6 |  | 4.6 ± 1.3 | 1.8 ± 1.8 |
| 1-triacontanol |  | C_30_ | 13.6 | 57.5 ± 6.4 | 54.2 ± 3.0 |  | 18.7 ± 5.4 | 7.4 ± 5.8 |
| **Total alcohols** | |  |  | **65.0 ± 6.0 A** | **65.64 ± 2.5 A** |  | **34.6 ± 8.1 B** | **26.1 ± 14.3B** |
| n-docosane | CH_3_(CH_2_)_n-2_CH_3_ | C_22_ | 11.1 | 1.5 ± 2.6 | 2.4 ± 1.5 |  | 5.9 ± 3.4 | 8.8 ± 4.2 |
| n-tricosane |  | C_23_ | 11.6 | *n.d.* | *n.d.* |  | 7.1 ± 5.9 | 11.0 ± 9.7 |
| n-pentacosane |  | C_25_ | 12.6 | 1.9 ± 2.5 | 1.8 ± 0.7 |  | 8.5 ± 3.3 | 9.8 ± 7.6 |
| n-nonacosane |  | C_29_ | 14.6 | 11.9 ± 2.4 | 6.8 ± 0.6 |  | 14.0 ± 5.7 | 5.0 ± 3.2 |
| **Total alkanes** | |  |  | **20.1 ± 6.4 A** | **13.64 ± 3.6 A** |  | **57.4 ± 10.2 B** | **58.7 ± 18.2 B** |
| 1-monopalmitin | CH_3_(CH_2_)_n-5_C(=O)OCH_2_CH(OH)CH_2_OH _-_ | C_19_ | 5.6 | *n.d.* | *n.d.* |  | 1.9 ± 1.2 | 2.5 ± 2.1 |
| 1-monostearin |  | C_21_ | 6.6 | 4.1 ± 1.5 | 9.5 ± 5.1 |  | 4.9 ± 2.9 | 7.0 ± 3.4 |
| **Total esters** | |  |  | **4.1 ± 1.5 A** | **9.5 ± 5.1 BC** |  | **6.8 ± 3.0 B** | **10.8 ± 2.4 C** |
| triacontanal | CH_3_(CH_2_)_n-2_C(=O)H | C_30_ | 13.6 | 10.8 ± 1.6 | 9.9 ± 0.7 |  | *n.d.* | *n.d.* |
| squalene^b^ | (C_5_H_8_)_6_ | C_30_ | 14.1 | 0.08 ± 0.02 | 1.8 ± 0.3 |  | 1.2 ± 0.5 | 2.3 ± 0.5 |
| Different letters in a row (A, B or C) correspond to statistically significant (*p*<0.05) differences between means of major chemical classes. | | | | | | | | |
| n.d., not detected. | | | | | | | | |
| ^a^Predicted data generated using the US Environmental Protection Agency’s EPISuite™.  ^b^ 2,6,10,15,19,23-hexametil-2,6,10,14,18,22-tetracosahexeno | | | | | | | | |

A

B


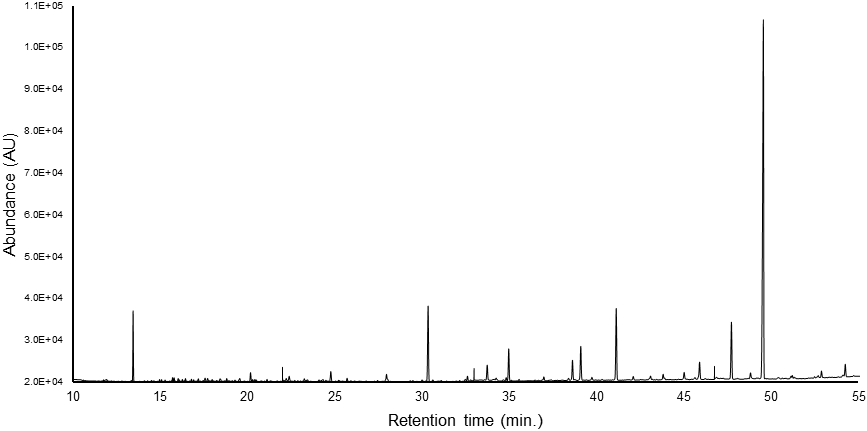

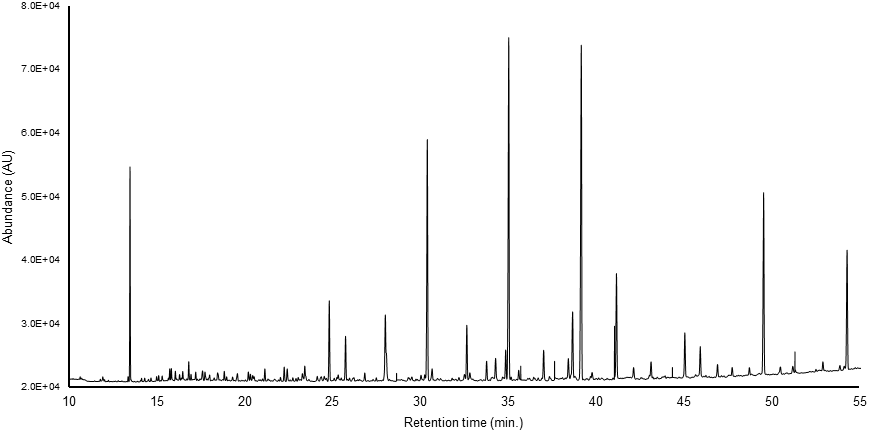


2

3

4

5

6

9

9

7

7

4

8

1

1

10

**Supplementary Figure 1.** GC-FID Chromatograms of the epicuticular waxes of the adaxial (A) and abaxial (B) surfaces of the *C. siliquastrum* leaf: (1) hexadecane; (2) 1-octadecanol*; (3) 1-eicosanol*; (4) tetracosane (internal standard); (5) 1-monopalmitin*; (6) 1-monostearin*; (7) nonacosane; (8) 1-triacontanal; (9) 1-triacontanol*; (10) tris(2,4-di-tert-butylphenyl) phosphate. Note: * TMS derivative.

|  | **Theoretical**  **retention index** | **Match** | **Reversed match** |
| --- | --- | --- | --- |
|  |  |  |  |
| 1-Octadecanol, TMS derivative | 2152 | 941 | 941 |
| Docosane* | 2200 | 900 | 902 |
| 1-Eicosanol* | 2281 | 828 | 880 |
| Tricosane* | 2300 | 870 | 871 |
| Pentacosane* | 2500 | 900 | 900 |
| Hexacosane* | 2600 | 935 | 940 |
| 2-Monostearin, 2TMS derivative | 2772 | 876 | 891 |
| Nonacosane* | 2900 | 865 | 865 |
| Hentriacontane* | 3100 | 900 | 905 |
| 1-Octacosanol, TMS derivative* | 3138 | 934 | 918 |
| Triacontanal* | 3251 | 824 | 864 |
| 1-Triacontanol, TMS derivative* | 3334 | 923 | 925 |
| *Compounds whose retention times and *m/z* spectra were compared to those of reference standards. | | | |

**Supplementary Table 2.** Chromatographic information regarding the most abundant compounds identified in *C. siliquastrum* leaf cuticular waxes.


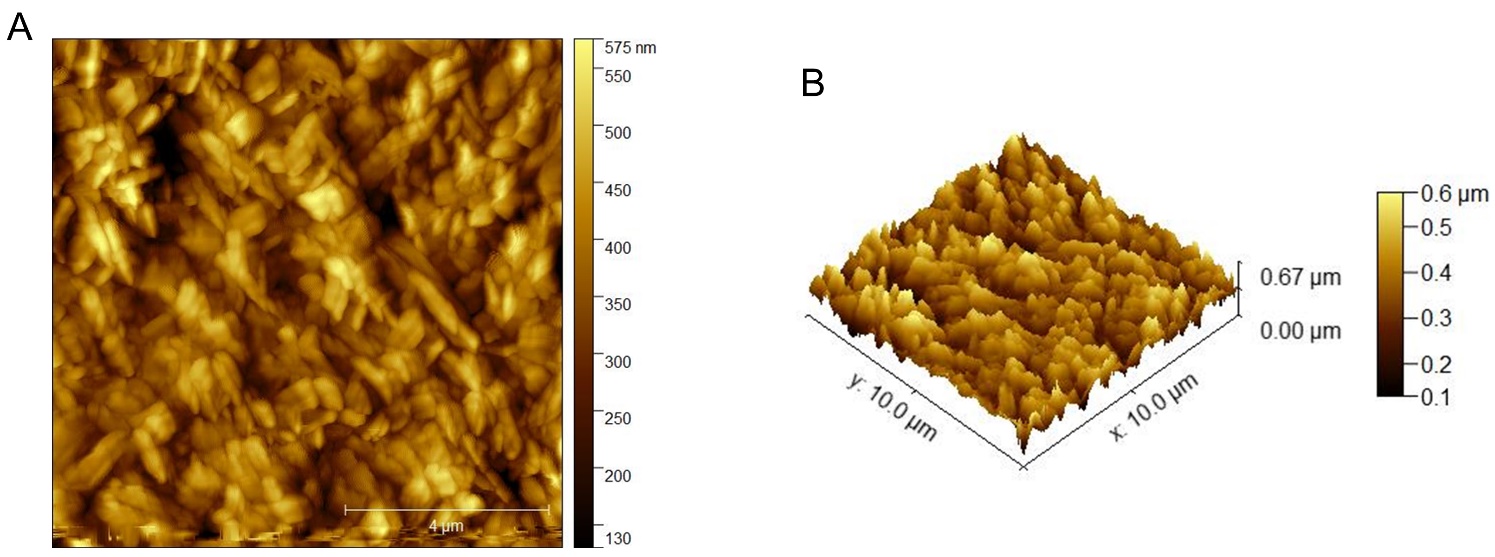


**Supplementary Figure 2.** AFM topographical (A) 2D and (B) 3D images of a representative region of the adaxial surface of a *C. siliquastrum* leaf.


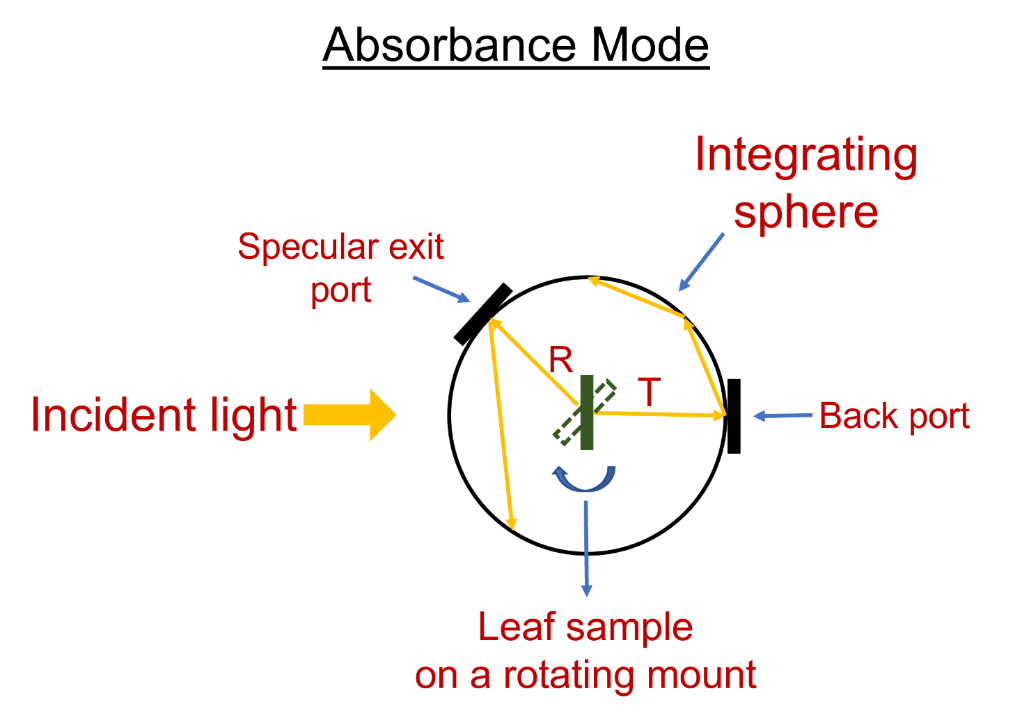


**Supplementary Figure 3.** Schematic representation of the spectrometer setup to measure absorbance of light of the *C. siliquastrum* leaf. The sample is placed on a rotating central mount. The angle of incident light is varied by rotating the leaf sample with respect to the incoming light. The integrating sphere collects both reflected (R) and transmitted (T) light and the absorbance (A) is then calculated as 1−R−T.
